# Treatment of municipal wastewater in full-scale on-site sand filter reduces BOD efficiently but does not reach requirements for nitrogen and phosphorus removal

**DOI:** 10.1007/s11356-017-8779-x

**Published:** 2017-03-18

**Authors:** Petteri Laaksonen, Aki Sinkkonen, Gennadi Zaitsev, Esa Mäkinen, Timo Grönroos, Martin Romantschuk

**Affiliations:** 1 Clewer Ltd, Biolinja 12, FI-20750 Turku, Finland; 2 Department of Environmental Sciences, University of Helsinki, Niemenkatu 73, FI-15140 Lahti, Finland; 3 Tekno-Forest Ltd, Kynttilätie 3, 11710 Riihimäki, Finland; 4 Turun Rakentajapalvelu, Puolaajankuja 13, 20660 Littoinen, Finland; 5 Institute of Environmental Sciences, Kazan Federal University, Kazan, 420008 Russia

**Keywords:** Household wastewater, Sand filter, Nitrogen removal, Phosphorus removal, BOD

## Abstract

A traditional sand filter for treatment of household wastewater was constructed in the fall of 2012 at Biolinja 12, Turku, Finland. Construction work was led and monitored by an authorized wastewater treatment consultant. The filter was placed on a field bordered by open ditches from all sides in order to collect excess rain and snowmelt waters. The filter was constructed and insulated from the environment so that all outflowing water was accounted for. Untreated, mainly municipal, wastewater from Varissuo suburb was pumped from a sewer separately via three septic tanks (volume = 1 m^3^ each) into the filters. Normally, wastewater was distributed to ground filters automatically according to pre-programmed schedule. Initially, the daily flow was 1200 L day^−1^ to reflect the average organic load of a household of five persons (load: ca 237 g day^−1^ BOD; 73 g day^−1^ total N; and 10.4 g day^−1^ total P). Later in the test, the flow rate was decreased first to 900 and then to 600 L day^−1^ to better reflect the average volume produced by five persons. Volumes of inlet wastewater as well as treated water were monitored by magnetic flow meters. Samples were withdrawn from the inlet water, from the water entering the filters after the third septic tank, and from the outflowing water. After an initial adaption time, the reductions in BOD and chemical oxygen demand were constantly between 92 and 98%, showing that the biological degradation process in the filters functioned optimally and clearly comply with the national and EU standards. The reduction in total nitrogen and total phosphorus, however, reached required levels only during the first months of testing, apparently when buildup of microbial biomass was still ongoing. After this initial period of 3 months showing satisfactory reduction levels, the reduction of total nitrogen varied between 5 and 25% and total phosphorus mostly between 50 and 65%. Nitrification was efficient in the filter, but as indicated by high nitrate levels and poor nitrogen reductions, denitrification was inefficient or absent. During the winter period, the temperature in the filter dropped to near freezing, but at all time points, the flow of water was unaffected by freezing. During snowmelt and heavy rain, occasional flooding was observed. Such situations may lead to dilution rather than purification of the wastewater. In conclusion, the sand filter tested worked well for reduction of the organic load in municipal wastewater but failed to sufficiently reduce nitrogen and phosphorus levels.

## Introduction

The current EU wastewater directive (91/271/EEC) has long been implemented in Finland, while new regulations in the Finnish government decree on treating domestic wastewater in areas outside sewer networks (209/2011; on-site decree; http://www.finlex.fi/fi/laki/alkup/2011/20110209) will gradually be implemented in Finland starting in the beginning of 2016, or based on new government plans, possibly delayed to 2019. Proper treatment of wastewaters will be then be enforced also in rural areas, including all households and vacation homes with running tap water. Initially, however, the decree will concern households that are equipped with water closet toilets and full bathroom, laundry, and kitchen facilities, generating one combined source of wastewater. The volume of wastewater from one family is therefore rather large, while the concentration of organic matter, nitrogen, phosphorus, etc. becomes quite low.

In cities and other densely populated areas, the municipal wastewater is treated with professionally constructed and up-kept wastewater treatment facilities that in Finland in most cases are based on biological active sludge processes. In normal use, these facilities do reach the requirements set by law, BOD reduction of 80% (90% in sensitive areas), total nitrogen removal of 30% (40% in sensitive areas), and total phosphorus removal of 60% (70% in sensitive areas), although occasional disturbances may occur. These facilities have specific separate phases for active aerobic degradation of the organic load, which is often the compartment where nitrification of ammonia is active. Converting the nitrate into nitrogen gas through denitrification is then active in a separate, anaerobic compartment. The heterotrophic bacteria active in denitrification do require organic substrates for proper function (Plascencia-Jatomea et al. 2015; Plüg et al. 2015), and, therefore, denitrification does not function as a last step in already cleaned, nitrate-rich water. Using elaborate recirculation systems, sufficient denitrification and, thereby, reductions in nitrogen emissions can be achieved in these facilities.

Small wastewater treatment units have relatively recently been introduced as a solution for one family home and other smaller wastewater-generating entities. Some of these are batch operated while others are continuously operated and based on biofilms forming on solid support. This type of treatment units have certain advantages in that the space needed is small. The minimum investment needed is limited, since relatively inexpensive units can be found on the market. It should, however, be kept in mind that minimizing the investment may lead to unsatisfactory results in the long run. Examples of this was seen in a controlled test performed in realistic condition using municipal wastewater (Weckström 2010). Some of the units tested had a satisfactory performance during the warm part of the year but dissatisfactory results in the winter, while other units performed poorly all year. In contrast, Vllpas and Santala (2007) found the performance of sequencing batch reactors as well as conventional sand filters to be satisfactory.

Because of uncertainty with these units, alternatives have been sought. Natural ground infiltration has long been used in the countryside and may be functional in combination with sedimentation tanks, in very scarcely populated areas (reviewed by Beal et al. 2005). Such traditional systems do, however, work best if used only for grey water (Dalahmeh et al. 2014a; Lehtoranta et al. 2014) when toilet waste is collected and treated separately.

Another option is to use specific constructed ground filtration units that have been studied to some extent (Lehtoranta et al. 2014). There are many varieties on the market, and the authorities publish specifications and recommendations for how these should be built properly. Simple solutions are also very competitive in price, while more elaborate units that include compartments for phosphorus precipitation, etc. are more expensive. A difficult question is, however, how to prove functionality indisputably. In some cases in Scandinavia (Vllpas and Santala 2007; Elmefors and Ljung 2013), monitoring has been performed in the environment close to such units, but it is next to impossible to take into account the rate of dilution into soil water, groundwater, surface runoff, etc. All that it tells is that the nutrient levels, bacterial counts, etc. in nearby water reservoirs do not exceed the levels stated in environmental regulations.

Sand filtration as a wastewater treatment system has been tested also rigorously but, in most cases, only in laboratory scale and often using synthetic wastewater (Healy et al. 2007; Dalahmeh et al. 2014b). Garcia et al. (2013) compared on-site septic treatment systems (STSs) with aerobic treatment systems (ATS) in warm climate conditions. They concluded that the ATS was better, and that the units did not benefit from further soil treatment. In studies performed in Nova Scotia, Canada, in conditions comparable to those in Finland, the researchers found that properly constructed on-site wastewater systems can provide adequate treatment (Bridson-Pateman et al. 2013). The systems tested were lateral flow sand filters with good performance with regard to most wastewater components, although total phosphorus removal rates were reported to decrease with time (Wilson et al. 2011), and with a high degree of nitrification but very little denitrification (Havard et al. 2008; Wilson et al. 2011).

The purpose of the present study was to test a full-scale ground filtration unit in cold climate conditions in a way that accounts for all incoming water from all possible sources and all outflowing water that has passed through the unit. Included in the inflow is then the wastewater, rainwater, and snowmelt in the spring, while all water leaving the unit is accounted for, monitored, sampled, and included in the calculation of environmental burden. The ground filtration unit was constructed using best available expertise and following authority recommendations.

Sampling points were planned to represent wastewater inflow, sedimentation tank outflow/sand filter unit inflow, distribution well (preceding sand infiltration), and collection well (ground unit outflow). Water retention time in the whole unit and its compartment were considered in the calculation of reductions of various wastewater components.

Of particular interest was to determine the faith of organic matter (BOD), total nitrogen (N), ammonia/nitrate ratio (NH_4_/NO_3_), and total phosphorus (P), but also, bacterial levels, pH, temperature and other factors were monitored.

## Materials and methods

**Fig. 1:**
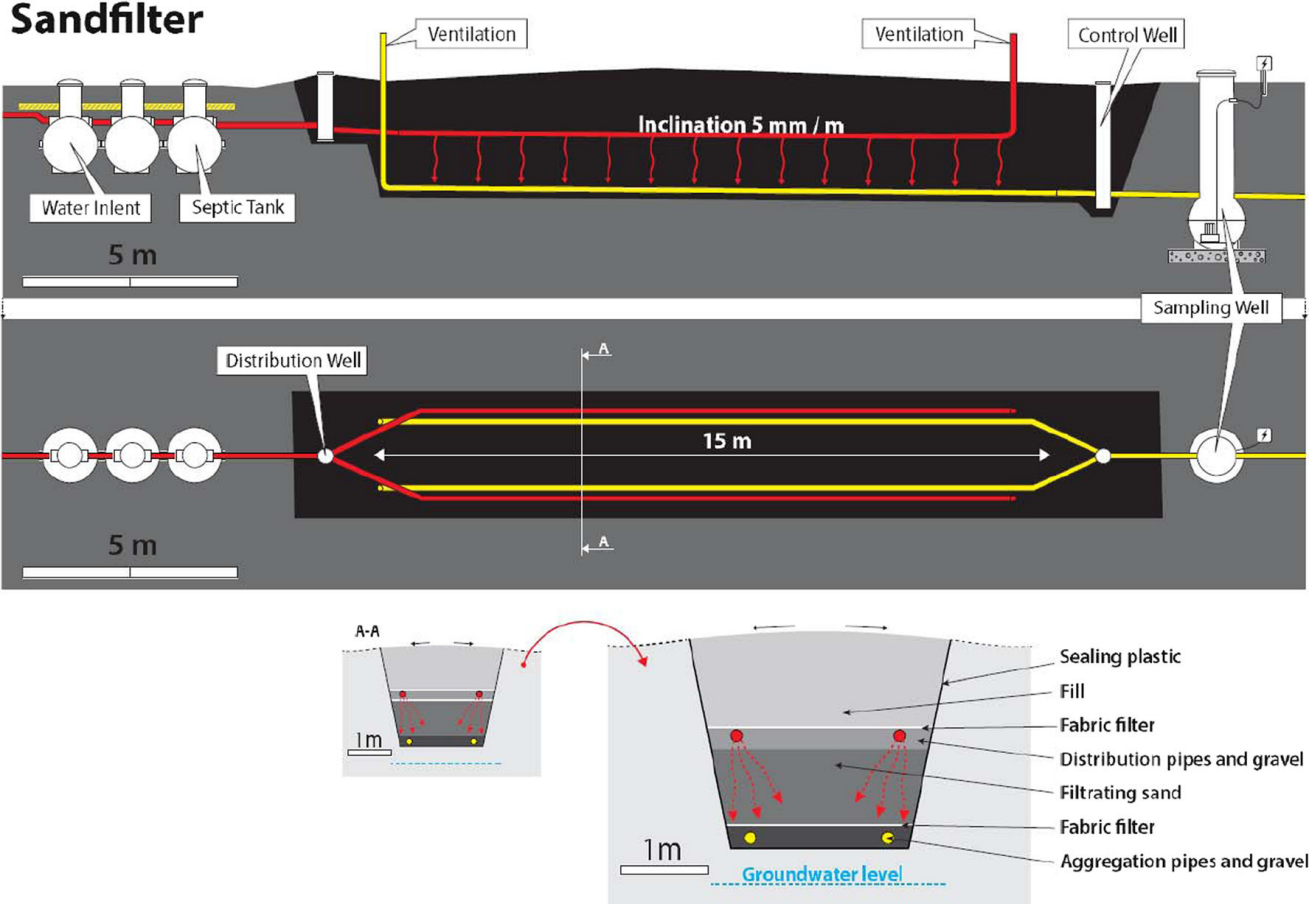
Graphic representation of the sand filter wastewater cleaning setup. Side view (*top panel*), from above (*middle panel*), cross section of the middle section (*bottom panel*). For clarity, the cross section is given in the same scale as the side view (*bottom left*) and enlarged (*bottom right*). The *black area* represents the sand filter isolated from the surrounding soil by a plastic lining

The ground filtration unit was built according to Finnish standards (Finlex 2011). The construction was carried out by Turun Rakentajapalvelu according to plans made by wastewater treatment consultant Timo Grönroos who also supervised the construction. The unit was placed in a field that was bordered by open ditches to collect excess rain and snowmelt waters. A dugout was made to fit three septic tanks, each with a volume of 1 m^3^, followed by the sand filtration unit built on top of a plastic sealing (Fig. 1). The plastic sealing guaranteed collection of all treated water as well as rain and snowmelt waters entering the unit. Before construction, long-term acceptance rate (LTAR) for the filtering sand was analyzed with Nyberg’s method (FANN, Järfälla, Sweden; Pell and Nyberg 1989). Sand with a three measurement average LTAR of 160 L m^−2^ day^−1^ was considered suitable and used. On top of the plastic seal, two perforated aggregation pipes were inserted in a layer of gravel. The upstream end of the aggregation pipes was led to the outside air to enable ventilation, while the other end was connected to a control well that was further connected to a sampling well, via which all water exiting the filtration unit had to pass. Filtrating sand was placed on top of a fabric filter as a 1-m layer. For feeding of wastewater, two stretches of perforated pipes were inserted with an inclination of 5 mm m^−1^ in a gravel layer on top of the filtrating sand at a depth of 50 cm under a layer of fill sand. The wastewater was fed into the first septic tank from which it traveled via the following tanks and via a distribution well into the distribution pipes. The far end of the distribution pipe was drawn to the soil surface to function as a ventilation inlet outside of feeding times.

### Wastewater source, type, and flow rates

**Fig. 2:**
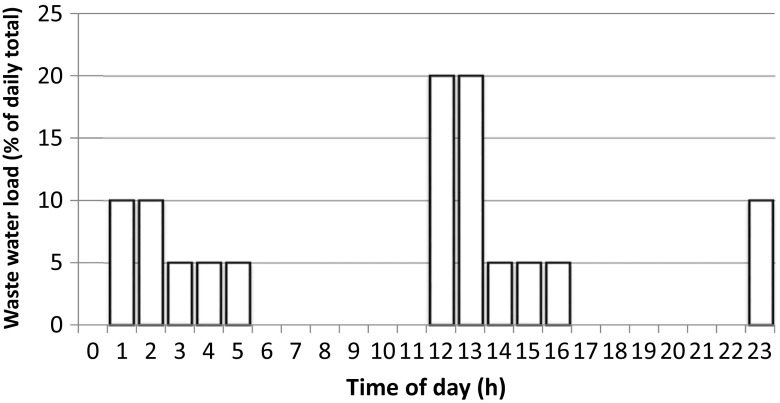
Distribution of wastewater load during the day. Dishwashing and laundry are assumed to be programmed for night hours, normal generation of kitchen and lavatory water during afternoon, and showering at bedtime. The volumes per day are presented in Table 2

Untreated, mainly municipal, wastewater from Varissuo suburb in Turku, South-Western Finland, was pumped from a sewer next to the research facility. During normal testing, wastewater was distributed to the sand filter automatically according to pre-programmed schedule roughly mimicking the water consumption in an average five-person family (Fig. 2; 35% during five nightly hours representing programmed washing machines, etc., 55% during five afternoon hours, and 10% during 1 h at bedtime). The daily load varied during different test periods (Table 1).

**Table 1:** Running parameters and sampling regimen in the ground filtration unit

Period (date)	Inflow (L day^−1^)	Samples	Duration (days)
December 12, 2012–September 12, 2013 (extended test period 1)	1200	25	280
September 24, 2013–January 28, 2014 (extended test period 2)	900	8	126
February 4–21, 2014 (test period 3; winter)	900	18	18
March 10–26, 2014 (test period 4; spring)	600	13	17
August 7–22, 2014 (test period 5; summer)	600	4	16

All outflow water passed via the control well into the sampling well after which it was returned to the sewer system. Since the surface of the fill covering the filter unit was uncovered, also rainwater and snowmelt entered the unit and was recovered at the outflow. The plastic sealing was installed both to stop wastewater from escaping the unit and to stop water from entering the unit from surrounding soil. The observed outflowing water volumes sometimes exceeded expectations, indicating that water diffusing from the surrounding areas was not completely stopped. Volumes of inlet wastewater as well as treated water were monitored by magnetic flow meters (MJK Automation, Nærum, Denmark). Water sampling was done from the sedimentation tanks, at the entry of the water into the inlet tubes (distribution well), at the control well, and at the sampling well representing the water outlet. Temperature of incoming untreated wastewater as well as sand temperature were continuously monitored. A sand temperature sensor was installed approximately 1 m from the surface. Temperature was measured at two levels inside the unit, in the sand and in the ventilation tube. The ambient temperature was also recorded and compared to those obtained from the local weather service.

### Chemical and microbiological analyses

Excluding retention time experiments, samples were taken with Bühler 1027 automatic samplers (Hach, Loveland, CO, USA). The samplers were programmed to take a 50-mL sample every 18 min during 24 h. Apparently due to retention, the hourly outflow was more dependent on weather conditions than on the programmed uneven inflow. Hach-Lange cuvette test kits were used for analyzing the chemical components in the water (Hach, Loveland, CO, USA). Bacteria were concentrated onto 0.45-μm filters that were incubated on mFC agar (Sigma-Aldrich, USA) plates for 24 h at 44.5 °C.

Test kits and measurement devices were as presented in Table 2.

**Table 2:** Methods for determining physical, chemical, and biological parameters in water samples

Analysis	Method
Biological oxygen demand (BOD_7ATU_)	Hach-Lange LCK 555/554
Chemical Oxygen Demand (COD_Cr_)	Hach-Lange LCK 114/314
Total nitrogen (N_tot_)	Hach-Lange LCK 138
Total phosphorus (P_tot_)	Hach-Lange LCK 349
Ammonium nitrogen (NH_4_-N)	Hach-Lange LCK 304
Nitrate nitrogen (NO_3_-N)	Hach-Lange LCK 339
Nitrite nitrogen (NO_2_-N)	Hach-Lange LCK 341
Chloride (Cl^−^)	Hach-Lange LCK 311
Potassium (K^+^)	Hach-Lange LCK 328 and ICP-MS
pH	Hach HQ411d meter + IntelliCAL PHC301 probe
Conductivity	Jenway 4071 portable meter
Thermotolerant fecal coliforms	Membrane filtration (0.45 μm )and incubation 24 h at 44.5 °C, mFC agar

During retention time tests, potassium was also measured by Atomic Absorption Spectrometry (AAS; Thermo, Waltham, MA, USA).

### Determination of hydraulic retention time

Prior to the intensive sampling periods, retention time tests were performed, and the results were taken into account when calculating reductions during the intensive sampling periods. The hydraulic retention time (HRT) in the sand filter was first studied by adding 1 kg of potassium nitrate (KNO_3_), 2 kg of potassium chloride (KCl), and 2 kg of calcium nitrate (CaNO_3_)_2_ into the last septic tank before the filter. During the HRT time experiment, wastewater was pumped into the system 1 m^3^ day^−1^. During this test, the feeding schedule was changed to pumping in wastewater every hour in order to maintain steady flow. This was done to limit the variables influencing the retention time calculations, bearing in mind that also the water inflow from the environment might obscure the calculations.

Cl^−^, K^+^, and NO_3_-N concentrations and conductivity in untreated and treated water were analyzed. Three control samples before and six samples after the addition were taken in order to monitor the retention of the ions in the sand filter. Samples were taken as spot samples from the distribution well (inlet of sand filter) and the control well (outlet).

In the second hydraulic retention time experiment, approximately 5 kg of KCl was dissolved into 20 L of water and added into the 15-L distribution well (Fig. 1). Wastewater was pumped at a constant rate equaling 1 m^3^ day^−1^. Samples were taken as mentioned. Cl^−^ and K^+^ concentrations and conductivity were analyzed. Three samples before and 20 samples after the addition were taken in order span the whole event.

During the intensive sampling periods (test periods 3–5) representing winter, spring, and summer conditions, respectively, reductions (%) in inlet vs. outlet loads of the whole system including the septic tanks were calculated using several different putative retention time alternatives of 24, 48, 72, and 120 h. As these did not differ statistically in reduction percentages, the 72-h scenario is used unless otherwise stated. The validity of this time lag is also supported by the retention time tests. Note, however, that particularly in the summer, the time lag may depend on precipitation and evaporation, while in the spring, snowmelt influences the outflow volumes considerably.

### Weather monitoring

**Fig. 3:**
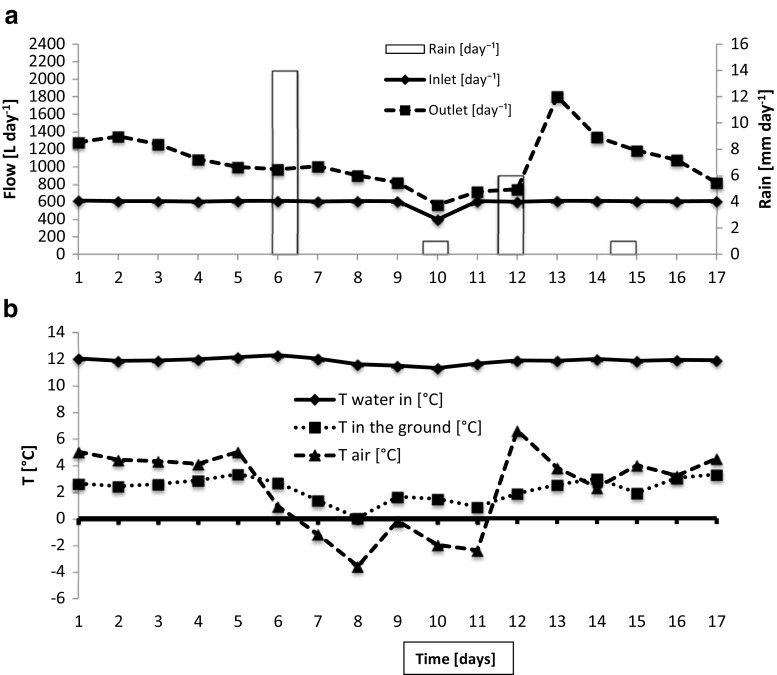
Basic parameters of test period 4. **a** Inlet and outlet water volumes and precipitation. **b** Temperature in inlet water, ground, and air. Period 4—spring (from March 10 to March 26, 2014) was chosen to illustrate the interdependence of temperature (snowmelt) and precipitation (rain) on the outflow volumes

Precipitation and ambient temperature were recorded locally (Fig. 3) and obtained from the Turku region weather service. Precipitation amounts and type (rain, snow) were obtained from the weather service.

### Running parameters during test periods 1–5

Wastewater feed was started December 12, 2012. Basic parameters of the test periods are presented in Table 2. In order to achieve a wastewater impurity load equivalent to five persons’ discharge, untreated wastewater was pumped into the filter at a rate of 1200 L day^−1^ during the extended test period 1. During the extended test period 2, wastewater feed was decreased to 900 L day^−1^, which is the maximum hydraulic flow capacity recommended for the unit. Aggregate samples were collected each day by automatic sampling every 18th minute. Of these daily aggregates, approximately three samples per month were picked for further analysis. From untreated wastewater (inflow) and treated waters (outflow), BOD_7ATU_, COD_Cr_, P_tot_, N_tot_, NH_4_-N, and NO_3_-N were analyzed.

During the extended experimental periods (periods 1 and 2), inflow and outflow samples were taken at the same hour. The steady input impurity levels entering the filtering unit from septic tank 3 enable valid impurity reduction levels to be calculated.

During the test period 3 (winter; February 4 to 21, 2014), the flow of untreated water was 900 L day^−1^ and sampling was done daily. If the total volume of the septic tanks is used, the calculated retention time in the septic tanks was 80 h. The specific sand filter retention time tests showed that the sand filter itself adds 8 h or more to the retention time, giving a theoretical total of 88 h, but the actual average retention time in the septic tanks is shortened by incomplete mixing in the tanks. An approximation of 72 h was used as the retention time for test period 3. The exact times vary also with the amount of rain, snowmelt, etc., but this single retention time was used for practical analysis-technical reasons. From inflow and outflow, COD_Cr_, N_tot_, P_tot_, NH_4_-N, NO_3_-N, and fecal coliforms were analyzed. Since the BOD/chemical oxygen demand (COD) ratio was found to be quite stable during the first run, the BOD_7_ values were calculated from the COD_Cr_ values.

Reduction in impurity load was calculated according to Eq. 1.

1 $$ \mathrm{Reduction}\%=\frac{\left({Load}_{\mathrm{in}-72\ \mathrm{h}}-{Load}_{\mathrm{out}}\right)}{Load_{\mathrm{in}-72\ \mathrm{h}}}\times 100\% $$

where *Load*_in – 72 h_ = impurity load into the system 72 h ago as per gram.

*Load*_out_ = impurity load out from the system as per gram.

For the test period 4 (spring, from March 10 to March 26, 2014), the flow of untreated water was set at 600 L day^−1^. Sampling was done daily, except for March 22 to 25. Analyses were as for the period 3. Due to lower flow rate, the calculated retention time for the whole unit was 120 h, which was taken into account in the reduction calculations.

Reduction in impurity load was calculated according to Eq. 2

2 $$ \mathrm{Reduction}\%=\frac{\left({Load}_{\mathrm{in}-120\ \mathrm{h}}-{Load}_{\mathrm{out}}\right)}{Load_{\mathrm{in}-120\ \mathrm{h}}}\times 100\% $$

Where *Load*_*i*n– 120 h_ = impurity load into the system 120 h ago as grams per day.

*Load*_out_ = impurity load out from the system as per gram.

The test period 5 (summer, August 7–22, 2014) repeated the running parameters and analysis schemes of test period 4 but was run during the warmer summer period.

The linear and non-linear regression lines were fit to the data as presented in respective figures.

## Results

### Physico-chemical parameters

The sand filter unit for wastewater treatment was in constant use from December 12, 2012. Only the inflow, and thereby load, and occasional amendments for retention time testing varied in a controlled manner.

The fluctuations in outflow volumes varied much more than the inflow volumes. These uncontrolled fluctuations were not only a result of rain and snowmelt but also to some degree evaporation during dry periods. Occasionally, the outflow volumes exceeded somewhat the expectations based on inflow and weather, suggesting a limited water diffusion from surrounding soil.

To illustrate the interdependence of outflow and temperature and precipitation, the results of period 4 (spring) are presented in detail. During the spring test period the ambient temperature varied considerably. During that period, the filtration unit was covered by snow that had started to melt, which showed as increased volumes of outflow water. Figure 3a shows inflow, outflow, and precipitation during test period 4. While the load was constant (except a dip on day 10), the outflow varied according to the fluctuations in outside temperature (Fig. 3b) which affected speed of snowmelt. The precipitation at time points days 6 and 10 was in the form of snow, while at day 12 and day 15, precipitation was sleet and water. The outflow volumes were directly influenced by the outside and the ground temperatures, so that the outflow during the first 7 days was 1.7–2.3 times as high as the inflow. As the outside temperature fell below freezing (day 7) and the ground temperature reached 0 °C 1 day later, the outflow fell close to the level of the inflow. On day 12, the temperature rose rapidly to 7 °C bringing also the outflow level with a 1-day lag to three times the inflow level as snow on top of the filter melted.

Inflow and outflow were recorded during all periods. In addition to period 4 (above), also during the other periods, the outflow corresponded to the inflow in a foreseeable manner and responded rapidly to rain during warm periods and to elevation of temperatures during cold periods (data not shown). In all cases, the load of different wastewater components was calculated from the strictly controlled inflow volume and its measured concentration. For calculating the real outflow load of each component not only the concentration but also the constantly monitored outflow volume, which varied according to weather conditions, was taken into account. Thus, the outflow load is always given as grams per time unit—not as concentration.

The temperature of the incoming water varied with the season but much less than the ambient temperature. During the coldest season—test period 3 (winter)—the incoming water temperature varied between 11 and 13 °C, while the sand filter temperature at 1-m depth was approximately 1 °C for most of the period, 1 day dropping down to −0.8 °C. At no time point did the water in the sand filter freeze, as evidenced by the fact that the outflow volumes always were at least at the level of the inflow.

The pH of the incoming wastewater was relatively stable, varying between pH 7.0 and 8.0, while the outflow pH range was from 6.0 to 7.5. Between the test runs and seasons, no significant differences were observed (data not shown). pH was therefore considered as a stable parameter when interpreting the results. The observed pH range is low enough not to cause evaporation of ammonia and high enough to enable nitrification.

During test period 5 (summer), ambient temperatures varied between 14 and 29 °C, while the inflow water was between 20 and 22 °C, and the sand filter between 14.5 and 20 °C. As in the case of the spring period (Fig. 3b), the ground temperature was a smoothed version of the ambient temperature curve. A higher amount of rain (50 mm during a 3-day period) occurring at the end of the period resulted in up to 25% elevated outflow with a 1-day delay. Smaller rain showers did not register on the outflow scale, whereas dry periods resulted, apparently as a result of evaporation, in an outflow that was up to 10% lower than the inflow (data not shown).

During the extended test periods (periods 1 and 2), daily sampling was not performed, but the interdependence of inflow, outflow, and environmental variables were the same as for period 4. The significance of these extended periods was in demonstrating long-term trends in the behavior of BOD/COD, nitrogen, and phosphorus which are presented in the following sections.

### Hydraulic retention time

The hydraulic retention time test was performed with a loading rate of 1 m^3^ day^−1^—close to the loading rate of test periods 1–3. The first test of hydraulic retention showed that the time for a liquid fraction (KCl, KNO_3_, CaNO_3_ solutions) to travel from the last of the three septic tanks to the outlet sampling point had started at the 5-h sampling and lasted beyond 30 h (data not shown). The second test consisted of a sudden high peak concentration of salt (KCl) added to the distribution well, thus entering the sand filter immediately. The retention times were followed by measuring separately concentrations of K^+^, Cl^−^, and conductivity. This time, the outlet salt concentration started to rise at the 8-hour sampling, reaching a maximum lasting from time 8 h to time 70 h. By calculating the outlet salt quantities in the outflow starting from the initial rise in concentration until the return to below a 30% rise and calculating the time for half of the salt to pass, the average retention time was found to be 30 h. This figure is not very exact, however, since the peak of high salt concentrations in the outlet was vide. Based on this average, the complete system, including the three 1-m^3^ septic tanks, had a theoretical retention time of 102 h, but since the volume of the septic tank is not mixed, the actual retention time is lower.

### BOD reduction

The organic load (BOD_7ATU_ g day^−1^) of the 1200 L day^−1^ inflow of wastewater during test period 1 varied between 145 and 335 with an average of 227 (*n* = 18), equaling an average concentration of 189 mg L^−1^. The outlet load (g day^−1^) varied between 2 and 23 g day^−1^ averaging 8 g day^−1^ (*n* = 18), which translates into a reduction efficiency of 97%. During extended period 2 with a flow rate 900 L day^−1^, the average incoming load of 164 g day^−1^ (ranging from 113 to 237) dropped to an average of 3 g day^−1^ (between 1 and 8) as the outgoing load, giving an average reduction of 98%.

During the short test periods, the reduction in organic load remained high. Test period 3 (winter) had an average incoming load that was somewhat higher, 183 g day^−1^ (between 145 and 217), while the outgoing load was 2.8–9.5 g day^−1^, resulting in a calculated reduction of 97%. Test period 4 (spring) with the flow rate of 600 L day^−1^ had an average incoming load of 97 g day^−1^ (between 64 and 118), while the outgoing load was 2.7 g day^−1^ (between 0.6 and 3.8) again with a reduction efficiency of 97%. During test period 5 (summer), the incoming load varied between 38 and 98 g day^−1^, while the outgoing loads were below 5 g. Even with the lower loads at the end of August 2014, the reduction stayed >96%.

The BOD reduction both as concentration (>97%) and, more importantly, as load (g day^−1^, 97%) was very high throughout the 20-month usage of the unit, clearly exceeding the requirements (minimum 80% or 90% for sensitive areas).

### Nitrogen and phosphorus removal

**Fig. 4:**
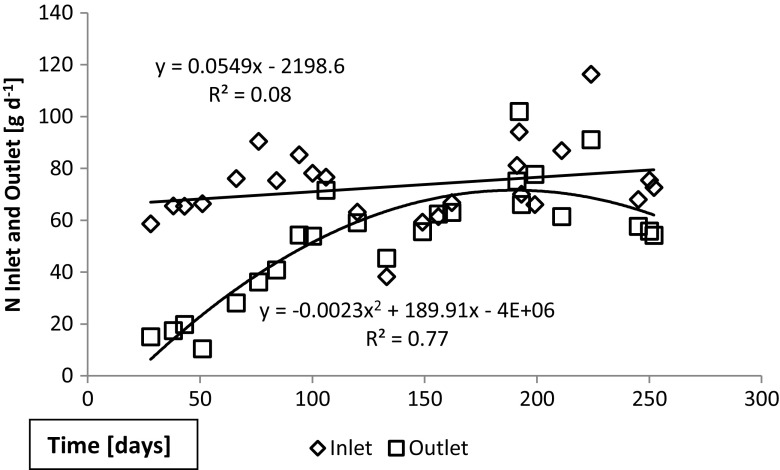
Total nitrogen quantities in inlet and outlet waters during the extended test period 1. The quantities of total nitrogen entering into and emerging from the wastewater treatment unit has been calculated from the N concentrations taking into account the constant inlet volumes and the outlet volumes which are affected by to environmental conditions such as rain and snowmelt

**Fig. 5:**
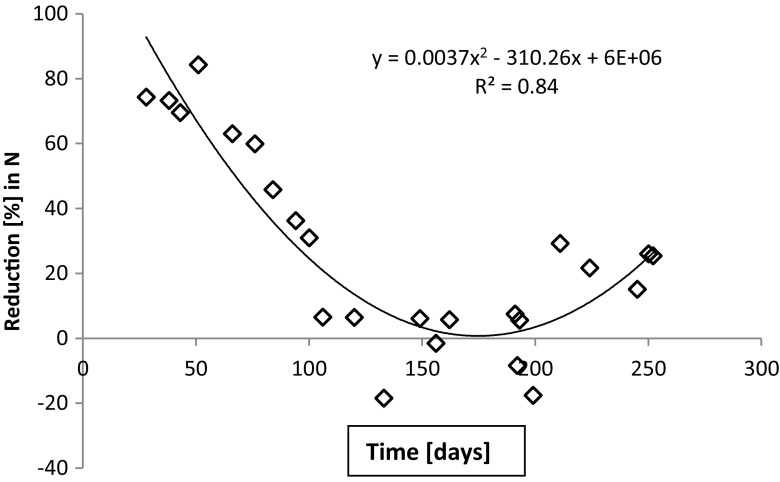
Reduction in total nitrogen quantities. When comparing inlet and outlet quantities for the extended period 1, the initially satisfactory reduction efficiency (%) worsens with time

**Fig. 6:**
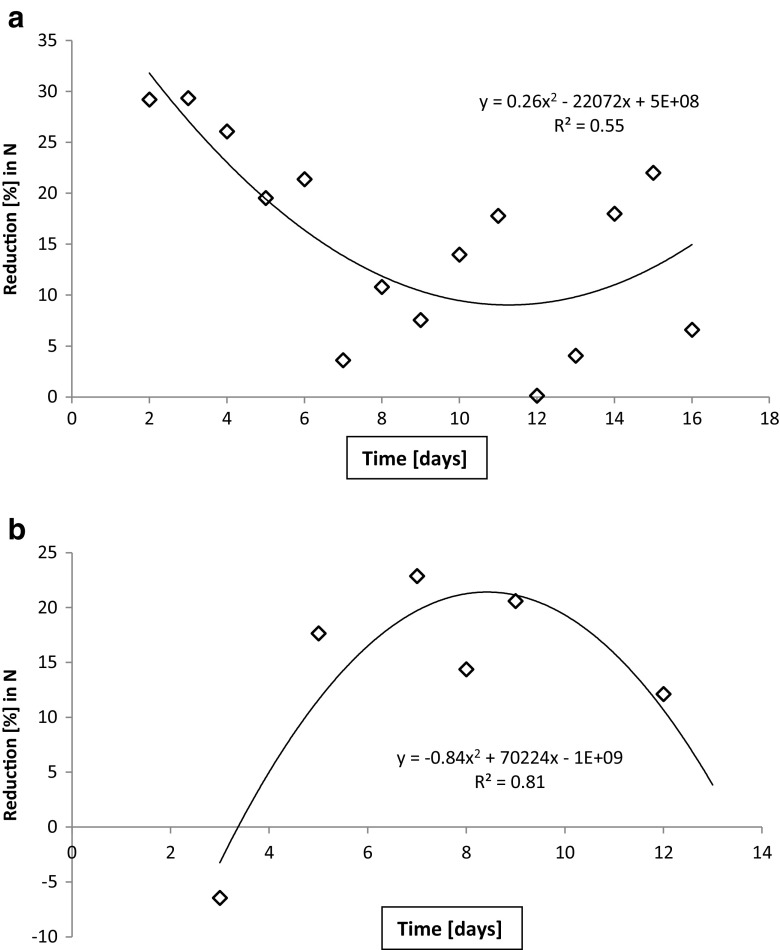
Reduction in total nitrogen quantities during short test periods 3 winter (**a**) and 5 summer (**b**). Reduction efficiency (%, 72-h lag) is below requirements at all time points

The load of total nitrogen was similar throughout the experimental periods. In case of the extended period 1, the total nitrogen load varied on a daily basis (38–116 g day^−1^), with an average of 73, while the N_tot_ in the outflow increased during the course of the test (Fig. 4). The reduction in total nitrogen concentrations was initially reaching the legislative thresholds of 30 (general) and 40% (sensitive areas), but for most of the time, the thresholds were not met (Fig. 5). The reduction stayed below 30% also during the second extended period (period 2) and the short test periods, of which periods 3 (winter) and 5 (summer) are presented in Fig. 6. Interestingly, during test period 4 (spring), the inlet varied from 15 to 27 g day^−1^ and the outlet 17–32 g day^−1^, possibly reflecting a flush out of built-up nitrogen reserves in the sand filter.

**Fig. 7:**
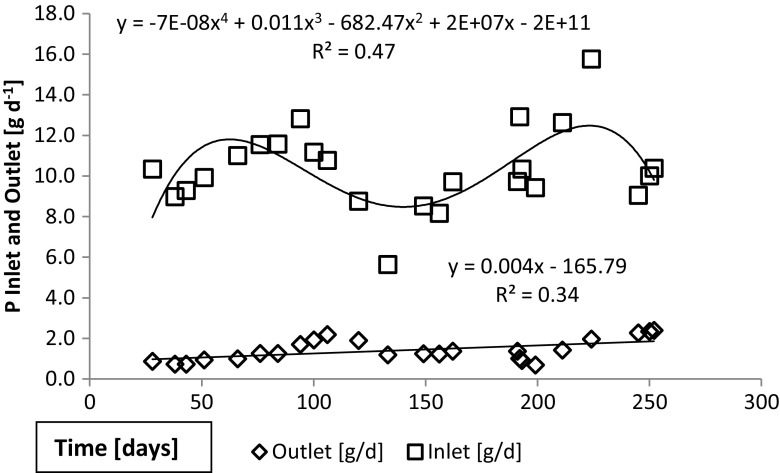
Total phosphorus quantities in inlet and outlet waters. The values for the extended period 1 are presented. During this, initial period the quantities in the outlet water was still quite small

**Fig. 8:**
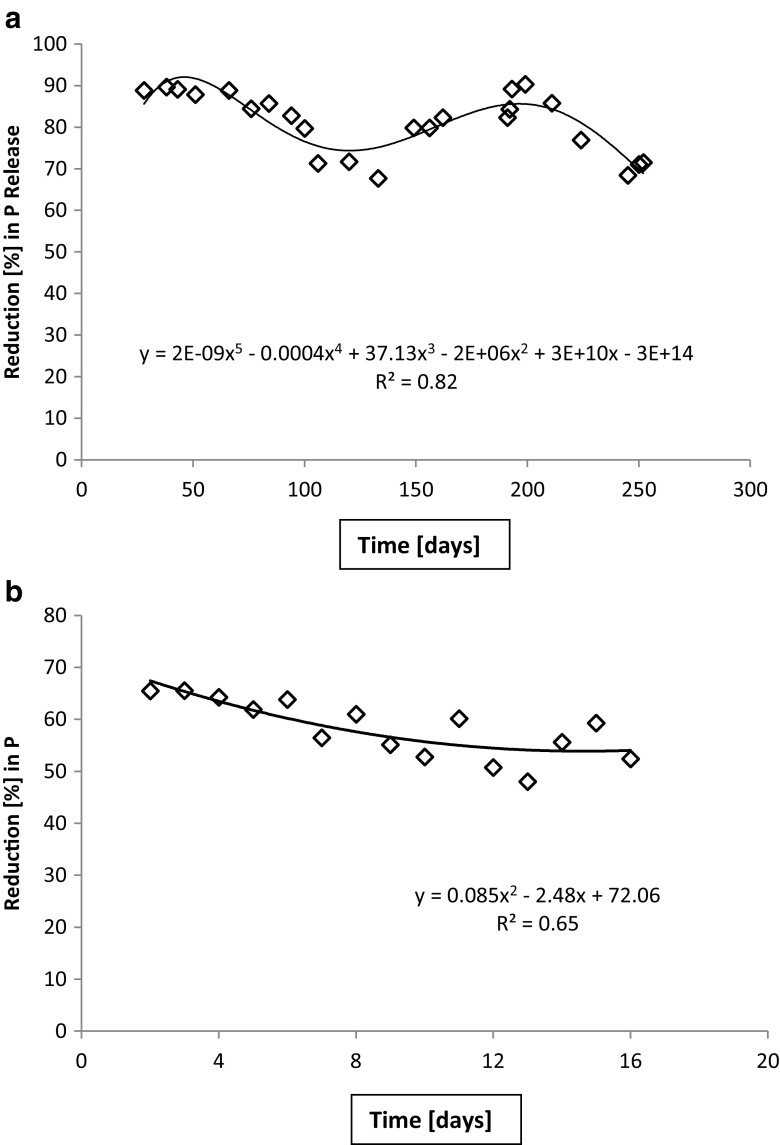
Reduction (%) in total phosphorus. Represented are the extended period 1 (**a**) and short period 3 winter February 6–22, 2014 (**b**)

During the extended period 1, the incoming load of total phosphorus varied between 6 and 16 g day^−1^ while the outlet stayed below 3 g day^−1^ (Fig. 7). As a result, reduction (%) in total phosphorus load was within legal limits, i.e., > 70%, for most, but not all of this long monitoring period (Fig. 8a). Furthermore, only occasional observations met the threshold for sensitive areas (85%). During the test period 3 (winter), the reduction was less than the required 70% throughout (Fig. 8b). During the test periods 4 (spring) and 5 (summer), the incoming load of total phosphorus was 3–5 g day^−1^ while the outflow varied from 1 to 3 g day^−1^. The reduction (%) varied from 51 to 60%, staying continuously below the required 70% (not shown).

### Nitrification

Most of the nitrogen present in the wastewater entering the septic tanks is in the form of urea or ammonia. During the extended period 1, the average ammonia load was 56 g day^−1^ (76% of N_tot_), while the nitrate concentration in the inflow was below detection. During test period 4 (spring), run with the lower inflow rate of 600 L day^−1^ the ammonia load was on average 18 g day^−1^ (75% of N_tot_). Similar results were obtained also for the other short test periods.

**Fig. 9:**
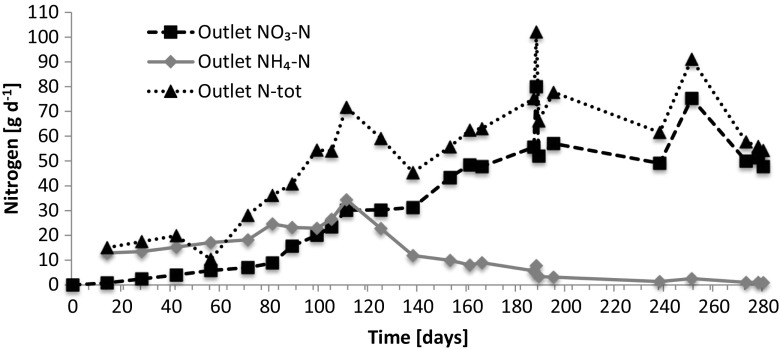
Forms of nitrogen in the outflow during extended period 1. Initially, a majority of the outflow nitrogen is in the form of ammonia, while nitrate starts to emerge after ca. 2 months. Simultaneously, the concentration of total nitrogen in the outflow also starts to rise. After ca. 6 months, the nitrogen emissions are mainly nitrate, and the total nitrogen concentration stays high throughout the test period. All nitrogen forms are presented as daily quantity of nitrogen in the outflow

During the extended period 1, an efficient nitrification process evolved during the first 4 to 6 months (Fig. 9), after which it stayed high (NO_3_/NH_4_ ratio >10) during all test periods. Low initial levels of total nitrogen coincide with a presumed buildup of microbial biomass and sorption during first 3 months of period 1.

### Reduction in bacterial counts

During test period 3 (winter), the fecal coliform colony forming units (cfu) were measured five times on different days in the incoming wastewater and in the outflow. The inflow density was relatively stable ranging from 1.3 × 10^9^ to 2.5 × 10^9^ 100 mL^−1^. The reduction at all time points was good, clearly exceeding the limit of 90% reduction. However, the outflow fecal coliform density was between 900 and 12,000 cfu 100 mL^−1^. The equivalent numbers for test period 4 (spring) was from 1.9 × 10^9^ to 6.7 × 10^9^ in the incoming and between 1600 and 3200 in the outflow, while the numbers for test period 5 (summer) were 5.2 × 10^9^ to 1.4 × 10^10^ and 100 to 1000, respectively, for the inflow and outflow. Thus, the summer wastewater contained the highest bacterial levels, but the reduction efficiency was best in these conditions.

During the spring test period, also the outflow of the third septic tank (inflow in sand filter) was analyzed. The counts were between 0.8 × 10^9^ and 2.3 × 10^9^, meaning that the reduction compared to the inflow was only between 32.3 and 87.5%. Septic tanks alone thus do not fulfill the requirements for hygienization.

## Discussion

Household wastewater has recently gained attention also in rural areas of Finland and countries with similar conditions. The legislation has been changed with a requirement that all households with running water should install an approved form of wastewater treatment. Details of the law as well as the implementation date are, however, still unclear. Lively debate goes on regarding functionality of different technical solutions, and, also, the implementation schedule has been pushed forward.

Many products intended for one-family homes, representing different kinds of batch, or continuous units, were introduced to the market during early 2000. Many of the brands were also tested and CE certified, but there was confusion regarding the load that some of these units could handle. Since price was one of the major selling arguments, this factor appears in some cases to have overshadowed reliable and stable functionality. When tested, many of these units performed suboptimally when the load was at the required upper limit, and also sudden peaks or longer pauses in the loading resulted in malfunction (Weckström 2010). Partly because of bad reputation of the small reactors, alternatives are sought for. Among those considered promising, sand filtration has gained attention and has been recommended by authorities also in Finland. This, however, has been done without proper, empirical knowledge on overall functionality in realistic, local environmental conditions. Although sand filtration has been studied in the USA and clear manuals have been published by the EPA (2002), full-scale realistic tests have not been performed in Finland or in similar conditions. The concept has been studied for mostly in laboratory condition and in small scale (e.g., Dalahmeh et al. 2012, 2014a, 2014b) or in pilot scale in laboratory conditions (Matikka and Heinonen-Tanski 2016). Most lab- or pilot-scale studies, however, aim at finding improvements and amendments to existing setups (e.g., Matikka and Heinonen-Tanski 2016) rather than determining how presently recommended setups function in practical conditions. Occasionally, field-scale tests have been done by monitoring the performance of installed units (Vllpas and Santala 2007). Estimation of load and inflow has mainly in these cases been based on theoretical calculations taking into account the number of users in the household. In order to fulfill strict scientific standards, all inflow volumes, concentrations, fluctuations, and physico-chemical parameters should be measures accurately, and the same applies for the outflow. Further, particular care should be taken to measure the outflow amounts of different components, and not only outflow concentrations, since volumes fluctuate in relation to precipitation, snowmelt, evaporation, etc. By sampling nearby water bodies, the element of dilution is uncontrolled.

For these reasons, we saw it important to perform the study presented here. Finnish law (209/2011) defines that a wastewater system for a one-family home must be able to handle a wastewater load of five people. Calculated as organic matter (BOD), nitrogen, and phosphorus, the loads per day are 250, 70, and 11 g, respectively. Calculating with a presumed water consumption of 120 L day^−1^ per person, this translates into the following concentrations: BOD: 417, N: 117, and P: 18 mg L^−1^. The wastewater used in this study, municipal wastewater from a residential area, was somewhat more dilute than the average household wastewater.

Trend lines in Figs. 4, 5, 6, 7, and 8 show the degree of reduction in nitrogen and phosphorus concentrations, even though polynomial functions in the figures could be substituted with simpler alternatives. These, however, would not change the fact that the tested sand filter system was incapable of limiting nitrogen emissions, while phosphorus concentrations were partly controlled. The incapability persisted for the whole 10-month monitoring period (Figs. 4, 7, and 8), and it was further supported during short periods of daily measurements (Figs. 5, 6, and 8). Reduction in levels of nitrogen can transiently be achieved through buildup of microbial biomass in the ground filtration unit. A more important long-term mechanism is, however, denitrification which has to be preceded by nitrification in ammonia-rich wastewater. After a lag period, nitrification was very efficient in the unit tested here, showing also that the aeration of the filtration unit was efficient and conditions aerobic. No denitrification could take place, apparently in part due to lack of anoxic conditions and in part to lack of substrates (low BOD) for the heterotrophic processes needed. The relative importance of these reasons cannot be assessed in this case. Suffice to say that efficient N removal by denitrification is impossible in the type of setup tested. Similar observations were recently reported by Garcia et al. (2013), who showed that an in essence anaerobic septic tank-based system left the nitrogen in the form of ammonia, while an aerobic treatment system transferred almost all the nitrogen into nitrate. Neither of the systems was efficient in removal of nitrogen from the water, compared to a municipal wastewater treatment system. Of the two on-site systems, only the aerobic one reduced the BOD levels, while the septic tank system is comparable to our system before the sand filter. Garcia et al. (2013) do point out that the water in systems they tested typically receives additional treatment by soil before discharge to the environment. The data presented here shows, however, that such soil treatment is inefficient in removing nitrogen from septic tank treated water. The discharge from our sand filter is instead comparable to the aerobically treated water studied by Garcia et al. (2013). In a laboratory-scale stratified sand filter column equipped with water recirculation, COD of dairy wastewater was reduced efficiently (99%) and also total nitrogen removal was good (86%) (Healy et al. 2007). Nitrification was efficient, since no ammonia was left in the water. In this case, apparently, the anoxic recirculation tank, where three quarters of the water was returned, allowed for efficient denitrification in the presence of carbon sources for the denitrifying heterotrophs. This notion is supported by the fact that an identical setup without recirculation only removed 27% of the total N (Rodgers et al. 2005).

Recently Matikka and Heinonen-Tanski (2016) reported that a pilot-scale setup including a biotite layer run for 54 weeks in lab conditions showed good results for BOD and bacterial density reduction, in agreement with our results. The reduction in levels of total phosphorus and total nitrogen reported by Matikka and Heinonen-Tanski (2016) were variable, in the beginning, reduction of nitrogen was poor and reduction of phosphorus was good, while towards the end of the run, the situation was the reversed. Although valuable, this test did not account for the effect of changing environmental conditions such as temperature fluctuations, precipitation, etc.

Wastewater treatment systems installed in the field are not as completely isolated from the environment by plastic lining as the units used in this study. Nutrient removal is likely to continue in native soil as the wastewater is further infiltrated. In Finland, the groundwater is, however, often very close to the surface and, therefore, very vulnerable.

Alternative and complementing methods for ensuring sufficient nutrient uptake have been tested, and some of these have been discussed by, e.g., Matikka and Heinonen-Tanski (2016). As groundwater levels are typically close to ground surface in Finland (see, e.g., http://wwwi3.ymparisto.fi/i3/tilanne/ENG/groundwater/LOS.htm), a wastewater treatment system that depends on additional native soil nutrient removal cannot be universally recommended but may sometimes be accepted based on a case-by-case evaluation taking in account local conditions.

Thus, using current recommendations for building a sand filter for wastewater treatment may result in a unit with inadequate removal of nitrogen and phosphorus despite good performance in removal of organic matter and microbes. This shortcoming might be amended by additional treatment units, e.g., for precipitation of phosphorus, but such units are currently not required by law.

## Conclusions

A wastewater treatment system based on three septic tanks followed by a sand filter that was built according to Finnish standards performed well with regard to removal of BOD during all seasons during the 2-year testing period.

At all tested periods following the run-in of 3 months, nitrogen removal was poor, far below the levels required by Finnish law and EU directives.

Phosphorus removal met the required level of 70% during most but not all of the extended test period 1. From day 250 onwards, the requirement was not met.

After the run-in period of 70 days, nitrification was very efficient and almost all nitrogen in the effluent was in the form of nitrate, whereas conditions for denitrification were not observed.

We conclude that septic tanks followed by ground filters are not alone suitable for household wastewater treatment in Finnish conditions if the water includes lavatory waters.
